# 4-Amino-1-(2-benzoyl-1-phenyl­eth­yl)-3-phenyl-1*H*-1,2,4-triazol-5(4*H*)-thione

**DOI:** 10.1107/S1600536810054553

**Published:** 2011-01-12

**Authors:** Xiao-qiu Song, Lin Ye, He-wen Wang

**Affiliations:** a School of Perfume and Aroma Technology, Shanghai Institute of Technology, Shanghai 200235, People’s Republic of China; b College of Chemistry and Applied Chemistry, Huanggang Normal University, Huanggang 438000, People’s Republic of China

## Abstract

In the title compound, C_23_H_20_N_4_OS, the two phenyl rings of the diphenyl­propanone fragment form a dihedral angle of 86.8 (1)°, and the third phenyl ring attached to the triazole ring is twisted from the latter at 40.1 (1)°. In the crystal, mol­ecules are paired into centrosymmetric dimers *via* pairs of inter­molecular N—H⋯O and N—H⋯S hydrogen bonds.

## Related literature

For the crystal structures of related 1,2,4-triazole-5(4*H*)-thione derivatives, see: Al-Tamimi *et al.* (2010 ▶); Fun *et al.* (2009 ▶); Tan *et al.* (2010 ▶); Wang *et al.* (2011 ▶).
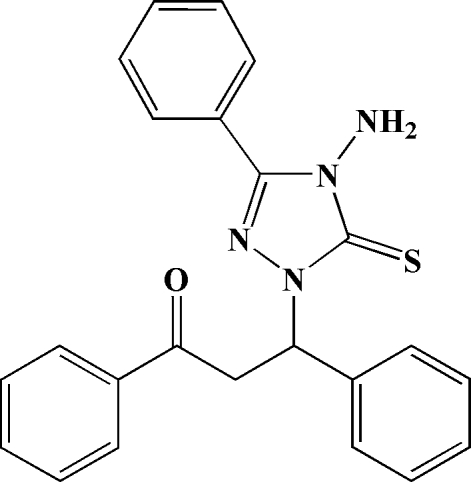


## Experimental

### 

#### Crystal data

C_23_H_20_N_4_OS
*M* *_r_* = 400.49
Triclinic, 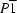

*a* = 9.4625 (19) Å
*b* = 11.340 (2) Å
*c* = 11.655 (2) Å
α = 111.80 (3)°
β = 111.01 (3)°
γ = 98.91 (3)°
*V* = 1022.4 (4) Å^3^
*Z* = 2
Mo *K*α radiation
μ = 0.18 mm^−1^
*T* = 113 K
0.20 × 0.18 × 0.10 mm

#### Data collection

Rigaku Saturn CCD area-detector diffractometer
Absorption correction: multi-scan (*CrystalClear*; Rigaku/MSC, 2005 ▶) *T*_min_ = 0.965, *T*_max_ = 0.982
9308 measured reflections
3590 independent reflections
2686 reflections with *I* > 2σ(*I*)
*R*_int_ = 0.039

#### Refinement

*R*[*F* ^2^ > 2σ(*F* ^2^)] = 0.037
*wR*(*F* ^2^) = 0.121
*S* = 1.04
3590 reflections
271 parameters
H atoms treated by a mixture of independent and constrained refinement
Δρ_max_ = 0.35 e Å^−3^
Δρ_min_ = −0.29 e Å^−3^

### 

Data collection: *CrystalClear* (Rigaku/MSC, 2005 ▶); cell refinement: *CrystalClear*; data reduction: *CrystalClear*; program(s) used to solve structure: *SHELXS97* (Sheldrick, 2008 ▶); program(s) used to refine structure: *SHELXL97* (Sheldrick, 2008 ▶); molecular graphics: *XP* in *SHELXTL* (Sheldrick, 2008 ▶); software used to prepare material for publication: *SHELXL97*.

## Supplementary Material

Crystal structure: contains datablocks global, I. DOI: 10.1107/S1600536810054553/cv5022sup1.cif

Structure factors: contains datablocks I. DOI: 10.1107/S1600536810054553/cv5022Isup2.hkl

Additional supplementary materials:  crystallographic information; 3D view; checkCIF report

## Figures and Tables

**Table 1:** Hydrogen-bond geometry (Å, °)

*D*—H⋯*A*	*D*—H	H⋯*A*	*D*⋯*A*	*D*—H⋯*A*
N4—H4*A*⋯O1^i^	0.91 (2)	2.39 (2)	2.873 (3)	114 (2)
N4—H4*B*⋯S1^i^	0.91 (2)	2.66 (2)	3.490 (2)	151 (2)

Symmetry code: (i) 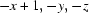.

**Fig. 1.**
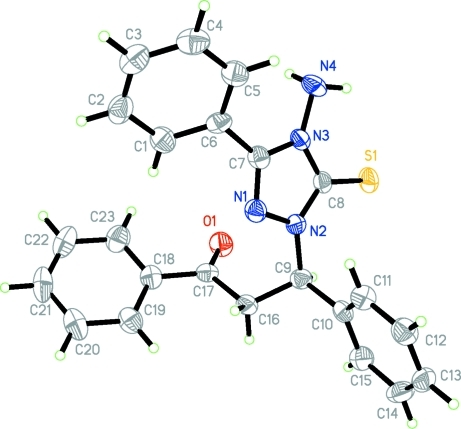
View of the molecule of (I) showing the atom-labelling scheme. Displacement ellipsoids are drawn at the 60% probability level.

## supplementary crystallographic information

### Comment

In continuation of structural study of 1,2,4-triazole-5(4*H*)-thione derivatives in our group (Wang *et al.*, 2011), we present here the crystal structure of the title compound, (I).

In (I) (Fig. 1), the bond lengths and angles are found to have normal values comparable with those observed in the related 1,2,4-triazole-5(4*H*)-thione derivatives (Al-Tamimi *et al.*, 2010; Fun *et al.*, 2009; Tan *et al.*, 2010; Wang *et al.*, 2011). There are three phenyl rings in the molecule. Phenyl ring A (C1—C6) attached in the triazole ring makes the dihedral angles of 61.0 (1) and 70.9 (1)° with the phenyl ring B (C10—C15) and C (C18—C23), respectively. Rings B and C form a dihedral angle of 86.8 (1)°.

Intermolecular N—H···S and N—H···O hydrogen bonds (Table 1) link the adjacent molecules into centrosymmetric dimers.

### Experimental

The title compound was synthesized by the reaction of the chalcone (2.0 mmol) with 4-amino-3-phenyl-4*H*-1,2,4-triazole-5-thiol (2.0 mmol) by refluxing in ethanol for 24 h. The reaction progress was monitored *via* TLC. The resulting precipitate was filtered off, washed with cold ethanol, dried and purified to give the target product as colorless solid in 87% yield. Crystals of (I) suitable for single-crystal X-ray analysis were grown by slow evaporation of a solution in chloroform-ethanol (1:1).

### Refinement

The H atoms attached to N atoms were located on a difference map, and isotropically refined using bond length restraint N—H = 0.91 (2) Å. C-bound H atoms were positioned geometrically (C—H = 0.95–1.00 Å), and refined as riding, with *U*_iso_(H) = 1.2-1.5*U*_eq_(C).

### Crystal data

**Table:**

C_23_H_20_N_4_OS	*Z* = 2
*M**_r_* = 400.49	*F*(000) = 420
Triclinic, *P*1	*D*_x_ = 1.301 Mg m^−^^3^
Hall symbol: -P 1	Mo *K*α radiation, λ = 0.71073 Å
*a* = 9.4625 (19) Å	Cell parameters from 3363 reflections
*b* = 11.340 (2) Å	θ = 2.1–27.8°
*c* = 11.655 (2) Å	µ = 0.18 mm^−^^1^
α = 111.80 (3)°	*T* = 113 K
β = 111.01 (3)°	Prism, colourless
γ = 98.91 (3)°	0.20 × 0.18 × 0.10 mm
*V* = 1022.4 (4) Å^3^	

### Data collection

**Table:**

Rigaku Saturn CCD area-detector diffractometer	3590 independent reflections
Radiation source: rotating anode	2686 reflections with *I* > 2σ(*I*)
multilayer	*R*_int_ = 0.039
Detector resolution: 7.31 pixels mm^-1^	θ_max_ = 25.0°, θ_min_ = 2.1°
φ and ω scans	*h* = −11→11
Absorption correction: multi-scan (*CrystalClear*; Rigaku/MSC, 2005)	*k* = −13→13
*T*_min_ = 0.965, *T*_max_ = 0.982	*l* = −13→12
9308 measured reflections	

### Refinement

**Table:**

Refinement on *F*^2^	Secondary atom site location: difference Fourier map
Least-squares matrix: full	Hydrogen site location: inferred from neighbouring sites
*R*[*F*^2^ > 2σ(*F*^2^)] = 0.037	H atoms treated by a mixture of independent and constrained refinement
*wR*(*F*^2^) = 0.121	*w* = 1/[σ^2^(*F*_o_^2^) + (0.071*P*)^2^ + 0.1941*P*] where *P* = (*F*_o_^2^ + 2*F*_c_^2^)/3
*S* = 1.04	(Δ/σ)_max_ = 0.001
3590 reflections	Δρ_max_ = 0.35 e Å^−^^3^
271 parameters	Δρ_min_ = −0.29 e Å^−^^3^
0 restraints	Extinction correction: *SHELXL97* (Sheldrick, 2008), Fc^*^=kFc[1+0.001xFc^2^λ^3^/sin(2θ)]^-1/4^
Primary atom site location: structure-invariant direct methods	Extinction coefficient: 0.107 (8)

### Special details

**Table:**

Geometry. All e.s.d.'s (except the e.s.d. in the dihedral angle between two l.s. planes) are estimated using the full covariance matrix. The cell e.s.d.'s are taken into account individually in the estimation of e.s.d.'s in distances, angles and torsion angles; correlations between e.s.d.'s in cell parameters are only used when they are defined by crystal symmetry. An approximate (isotropic) treatment of cell e.s.d.'s is used for estimating e.s.d.'s involving l.s. planes.
Refinement. Refinement of *F*^2^ against ALL reflections. The weighted *R*-factor *wR* and goodness of fit *S* are based on *F*^2^, conventional *R*-factors *R* are based on *F*, with *F* set to zero for negative *F*^2^. The threshold expression of *F*^2^ > σ(*F*^2^) is used only for calculating *R*-factors(gt) *etc*. and is not relevant to the choice of reflections for refinement. *R*-factors based on *F*^2^ are statistically about twice as large as those based on *F*, and *R*- factors based on ALL data will be even larger.

### Fractional atomic coordinates and isotropic or equivalent isotropic displacement parameters (Å^2^)

**Table:**

	*x*	*y*	*z*	*U*_iso_*/*U*_eq_	
S1	0.54562 (6)	−0.11721 (5)	0.13685 (5)	0.02158 (19)	
O1	0.45937 (17)	0.25822 (14)	0.24735 (14)	0.0237 (4)	
N1	0.82240 (19)	0.25821 (16)	0.32190 (16)	0.0182 (4)	
N2	0.72008 (19)	0.15086 (16)	0.31246 (16)	0.0163 (4)	
N3	0.75632 (19)	0.07328 (15)	0.13230 (15)	0.0159 (4)	
N4	0.7447 (2)	−0.01242 (17)	0.00269 (17)	0.0213 (4)	
H4A	0.703 (2)	−0.0962 (13)	−0.010 (2)	0.036 (7)*	
H4B	0.675 (2)	0.005 (2)	−0.062 (2)	0.055 (9)*	
C1	0.9302 (2)	0.4216 (2)	0.2059 (2)	0.0231 (5)	
H1	0.8614	0.4514	0.2443	0.028*	
C2	1.0231 (3)	0.5072 (2)	0.1812 (2)	0.0252 (5)	
H2	1.0168	0.5949	0.2010	0.030*	
C3	1.1260 (2)	0.4637 (2)	0.1269 (2)	0.0257 (5)	
H3	1.1913	0.5223	0.1111	0.031*	
C4	1.1330 (3)	0.3347 (2)	0.0962 (2)	0.0260 (5)	
H4	1.2038	0.3059	0.0600	0.031*	
C5	1.0376 (2)	0.2475 (2)	0.1179 (2)	0.0221 (5)	
H5	1.0408	0.1586	0.0943	0.026*	
C6	0.9371 (2)	0.2915 (2)	0.17470 (19)	0.0176 (4)	
C7	0.8422 (2)	0.20849 (19)	0.21041 (19)	0.0176 (5)	
C8	0.6737 (2)	0.03559 (19)	0.19571 (19)	0.0160 (4)	
C9	0.6600 (2)	0.17172 (19)	0.41591 (19)	0.0178 (5)	
H9	0.5473	0.1088	0.3687	0.021*	
C10	0.7610 (2)	0.14453 (18)	0.53269 (19)	0.0176 (4)	
C11	0.9151 (2)	0.1418 (2)	0.5597 (2)	0.0222 (5)	
H11	0.9606	0.1555	0.5032	0.027*	
C12	1.0040 (3)	0.1191 (2)	0.6688 (2)	0.0245 (5)	
H12	1.1093	0.1169	0.6861	0.029*	
C13	0.9392 (3)	0.0995 (2)	0.7523 (2)	0.0237 (5)	
H13	0.9991	0.0824	0.8259	0.028*	
C14	0.7863 (3)	0.1051 (2)	0.7281 (2)	0.0250 (5)	
H14	0.7426	0.0939	0.7866	0.030*	
C15	0.6970 (2)	0.1268 (2)	0.6188 (2)	0.0215 (5)	
H15	0.5921	0.1297	0.6023	0.026*	
C16	0.6580 (2)	0.31677 (19)	0.47288 (19)	0.0187 (5)	
H16A	0.6083	0.3303	0.5364	0.022*	
H16B	0.7697	0.3799	0.5269	0.022*	
C17	0.5659 (2)	0.3486 (2)	0.35832 (19)	0.0174 (4)	
C18	0.6095 (2)	0.4905 (2)	0.3827 (2)	0.0188 (5)	
C19	0.6976 (2)	0.5982 (2)	0.5161 (2)	0.0229 (5)	
H19	0.7328	0.5816	0.5941	0.028*	
C20	0.7336 (3)	0.7291 (2)	0.5348 (2)	0.0278 (5)	
H20	0.7929	0.8020	0.6258	0.033*	
C21	0.6842 (3)	0.7544 (2)	0.4223 (3)	0.0298 (6)	
H21	0.7084	0.8445	0.4361	0.036*	
C22	0.5990 (3)	0.6483 (2)	0.2889 (2)	0.0317 (6)	
H22	0.5672	0.6657	0.2114	0.038*	
C23	0.5603 (3)	0.5168 (2)	0.2688 (2)	0.0261 (5)	
H23	0.5003	0.4443	0.1775	0.031*	

### Atomic displacement parameters (Å^2^)

**Table:**

	*U*^11^	*U*^22^	*U*^33^	*U*^12^	*U*^13^	*U*^23^
S1	0.0236 (3)	0.0133 (3)	0.0240 (3)	0.0040 (2)	0.0089 (2)	0.0076 (2)
O1	0.0232 (8)	0.0199 (8)	0.0169 (7)	0.0047 (6)	0.0019 (6)	0.0057 (6)
N1	0.0185 (9)	0.0149 (9)	0.0190 (9)	0.0036 (7)	0.0084 (7)	0.0066 (7)
N2	0.0188 (9)	0.0134 (8)	0.0154 (8)	0.0037 (7)	0.0078 (7)	0.0061 (7)
N3	0.0171 (9)	0.0136 (8)	0.0144 (8)	0.0062 (7)	0.0056 (7)	0.0049 (7)
N4	0.0235 (10)	0.0178 (9)	0.0152 (9)	0.0066 (8)	0.0075 (8)	0.0017 (7)
C1	0.0231 (11)	0.0187 (11)	0.0211 (11)	0.0038 (9)	0.0079 (9)	0.0061 (9)
C2	0.0282 (12)	0.0217 (11)	0.0192 (11)	0.0033 (10)	0.0051 (10)	0.0106 (9)
C3	0.0215 (11)	0.0267 (12)	0.0212 (11)	−0.0007 (9)	0.0052 (9)	0.0114 (9)
C4	0.0206 (11)	0.0295 (12)	0.0226 (11)	0.0032 (10)	0.0104 (10)	0.0084 (10)
C5	0.0217 (11)	0.0198 (11)	0.0170 (10)	0.0039 (9)	0.0057 (9)	0.0052 (9)
C6	0.0154 (10)	0.0195 (10)	0.0105 (9)	0.0006 (8)	0.0029 (8)	0.0049 (8)
C7	0.0161 (10)	0.0146 (10)	0.0158 (10)	0.0026 (8)	0.0045 (8)	0.0046 (8)
C8	0.0166 (10)	0.0142 (10)	0.0161 (10)	0.0064 (8)	0.0057 (9)	0.0070 (8)
C9	0.0185 (10)	0.0168 (10)	0.0156 (10)	0.0038 (8)	0.0075 (9)	0.0062 (8)
C10	0.0200 (10)	0.0105 (9)	0.0166 (10)	0.0027 (8)	0.0068 (9)	0.0032 (8)
C11	0.0235 (11)	0.0216 (11)	0.0215 (11)	0.0076 (9)	0.0106 (9)	0.0096 (9)
C12	0.0238 (11)	0.0226 (11)	0.0225 (11)	0.0090 (9)	0.0078 (10)	0.0080 (9)
C13	0.0308 (12)	0.0195 (11)	0.0153 (10)	0.0067 (9)	0.0053 (10)	0.0082 (9)
C14	0.0314 (12)	0.0247 (11)	0.0183 (11)	0.0041 (10)	0.0130 (10)	0.0098 (9)
C15	0.0206 (11)	0.0191 (10)	0.0216 (11)	0.0032 (9)	0.0090 (9)	0.0081 (9)
C16	0.0213 (10)	0.0152 (10)	0.0158 (10)	0.0036 (8)	0.0079 (9)	0.0048 (8)
C17	0.0177 (10)	0.0196 (10)	0.0142 (10)	0.0065 (9)	0.0074 (9)	0.0069 (8)
C18	0.0143 (10)	0.0194 (11)	0.0241 (11)	0.0062 (8)	0.0097 (9)	0.0103 (9)
C19	0.0196 (11)	0.0195 (11)	0.0253 (11)	0.0043 (9)	0.0094 (9)	0.0077 (9)
C20	0.0234 (11)	0.0180 (11)	0.0388 (13)	0.0067 (9)	0.0149 (10)	0.0094 (10)
C21	0.0252 (12)	0.0222 (12)	0.0519 (15)	0.0120 (10)	0.0196 (11)	0.0232 (11)
C22	0.0323 (13)	0.0343 (13)	0.0386 (13)	0.0124 (11)	0.0151 (11)	0.0273 (11)
C23	0.0238 (11)	0.0267 (12)	0.0271 (12)	0.0071 (10)	0.0089 (10)	0.0149 (10)

### Geometric parameters (Å, °)

**Table:**

S1—C8	1.677 (2)	C10—C11	1.386 (3)
O1—C17	1.225 (2)	C10—C15	1.400 (3)
N1—C7	1.305 (3)	C11—C12	1.391 (3)
N1—N2	1.375 (2)	C11—H11	0.9500
N2—C8	1.351 (2)	C12—C13	1.385 (3)
N2—C9	1.468 (3)	C12—H12	0.9500
N3—C8	1.373 (3)	C13—C14	1.387 (3)
N3—C7	1.374 (3)	C13—H13	0.9500
N3—N4	1.413 (2)	C14—C15	1.387 (3)
N4—H4A	0.91 (2)	C14—H14	0.9500
N4—H4B	0.91 (2)	C15—H15	0.9500
C1—C2	1.387 (3)	C16—C17	1.514 (3)
C1—C6	1.400 (3)	C16—H16A	0.9900
C1—H1	0.9500	C16—H16B	0.9900
C2—C3	1.396 (3)	C17—C18	1.488 (3)
C2—H2	0.9500	C18—C19	1.396 (3)
C3—C4	1.389 (3)	C18—C23	1.400 (3)
C3—H3	0.9500	C19—C20	1.385 (3)
C4—C5	1.389 (3)	C19—H19	0.9500
C4—H4	0.9500	C20—C21	1.379 (3)
C5—C6	1.395 (3)	C20—H20	0.9500
C5—H5	0.9500	C21—C22	1.388 (3)
C6—C7	1.471 (3)	C21—H21	0.9500
C9—C10	1.527 (3)	C22—C23	1.386 (3)
C9—C16	1.534 (3)	C22—H22	0.9500
C9—H9	1.0000	C23—H23	0.9500
			
C7—N1—N2	104.69 (16)	C10—C11—C12	120.6 (2)
C8—N2—N1	113.11 (16)	C10—C11—H11	119.7
C8—N2—C9	126.58 (17)	C12—C11—H11	119.7
N1—N2—C9	120.00 (15)	C13—C12—C11	120.2 (2)
C8—N3—C7	109.01 (16)	C13—C12—H12	119.9
C8—N3—N4	124.81 (16)	C11—C12—H12	119.9
C7—N3—N4	126.05 (17)	C12—C13—C14	119.66 (19)
N3—N4—H4A	104.0 (15)	C12—C13—H13	120.2
N3—N4—H4B	106.7 (17)	C14—C13—H13	120.2
H4A—N4—H4B	111.0 (14)	C15—C14—C13	120.3 (2)
C2—C1—C6	120.4 (2)	C15—C14—H14	119.9
C2—C1—H1	119.8	C13—C14—H14	119.9
C6—C1—H1	119.8	C14—C15—C10	120.3 (2)
C1—C2—C3	119.5 (2)	C14—C15—H15	119.9
C1—C2—H2	120.2	C10—C15—H15	119.9
C3—C2—H2	120.2	C17—C16—C9	112.01 (16)
C4—C3—C2	120.0 (2)	C17—C16—H16A	109.2
C4—C3—H3	120.0	C9—C16—H16A	109.2
C2—C3—H3	120.0	C17—C16—H16B	109.2
C3—C4—C5	120.7 (2)	C9—C16—H16B	109.2
C3—C4—H4	119.6	H16A—C16—H16B	107.9
C5—C4—H4	119.6	O1—C17—C18	121.08 (18)
C4—C5—C6	119.5 (2)	O1—C17—C16	120.19 (18)
C4—C5—H5	120.3	C18—C17—C16	118.73 (17)
C6—C5—H5	120.3	C19—C18—C23	119.19 (19)
C5—C6—C1	119.83 (19)	C19—C18—C17	121.70 (18)
C5—C6—C7	122.41 (19)	C23—C18—C17	119.11 (18)
C1—C6—C7	117.70 (18)	C20—C19—C18	120.1 (2)
N1—C7—N3	110.27 (18)	C20—C19—H19	120.0
N1—C7—C6	122.88 (17)	C18—C19—H19	120.0
N3—C7—C6	126.76 (17)	C21—C20—C19	120.5 (2)
N2—C8—N3	102.87 (16)	C21—C20—H20	119.7
N2—C8—S1	130.11 (16)	C19—C20—H20	119.7
N3—C8—S1	127.01 (14)	C20—C21—C22	120.1 (2)
N2—C9—C10	112.15 (16)	C20—C21—H21	120.0
N2—C9—C16	107.96 (16)	C22—C21—H21	120.0
C10—C9—C16	111.00 (16)	C23—C22—C21	120.0 (2)
N2—C9—H9	108.6	C23—C22—H22	120.0
C10—C9—H9	108.6	C21—C22—H22	120.0
C16—C9—H9	108.6	C22—C23—C18	120.2 (2)
C11—C10—C15	118.93 (18)	C22—C23—H23	119.9
C11—C10—C9	122.58 (18)	C18—C23—H23	119.9
C15—C10—C9	118.44 (18)		
			
C7—N1—N2—C8	−1.1 (2)	C8—N2—C9—C16	−145.64 (18)
C7—N1—N2—C9	−175.02 (17)	N1—N2—C9—C16	27.4 (2)
C6—C1—C2—C3	−1.1 (3)	N2—C9—C10—C11	18.4 (3)
C1—C2—C3—C4	1.0 (3)	C16—C9—C10—C11	−102.5 (2)
C2—C3—C4—C5	0.4 (3)	N2—C9—C10—C15	−164.09 (17)
C3—C4—C5—C6	−1.8 (3)	C16—C9—C10—C15	75.1 (2)
C4—C5—C6—C1	1.6 (3)	C15—C10—C11—C12	1.3 (3)
C4—C5—C6—C7	−175.21 (18)	C9—C10—C11—C12	178.81 (18)
C2—C1—C6—C5	−0.2 (3)	C10—C11—C12—C13	−0.3 (3)
C2—C1—C6—C7	176.77 (18)	C11—C12—C13—C14	−1.1 (3)
N2—N1—C7—N3	−0.5 (2)	C12—C13—C14—C15	1.5 (3)
N2—N1—C7—C6	176.48 (17)	C13—C14—C15—C10	−0.5 (3)
C8—N3—C7—N1	1.8 (2)	C11—C10—C15—C14	−0.9 (3)
N4—N3—C7—N1	177.66 (16)	C9—C10—C15—C14	−178.48 (17)
C8—N3—C7—C6	−175.02 (18)	N2—C9—C16—C17	53.6 (2)
N4—N3—C7—C6	0.9 (3)	C10—C9—C16—C17	176.93 (16)
C5—C6—C7—N1	140.4 (2)	C9—C16—C17—O1	26.2 (3)
C1—C6—C7—N1	−36.5 (3)	C9—C16—C17—C18	−152.79 (18)
C5—C6—C7—N3	−43.1 (3)	O1—C17—C18—C19	162.63 (19)
C1—C6—C7—N3	139.9 (2)	C16—C17—C18—C19	−18.4 (3)
N1—N2—C8—N3	2.1 (2)	O1—C17—C18—C23	−17.1 (3)
C9—N2—C8—N3	175.54 (16)	C16—C17—C18—C23	161.87 (18)
N1—N2—C8—S1	−177.12 (14)	C23—C18—C19—C20	0.8 (3)
C9—N2—C8—S1	−3.6 (3)	C17—C18—C19—C20	−178.92 (18)
C7—N3—C8—N2	−2.2 (2)	C18—C19—C20—C21	−0.5 (3)
N4—N3—C8—N2	−178.20 (15)	C19—C20—C21—C22	−0.7 (3)
C7—N3—C8—S1	176.97 (14)	C20—C21—C22—C23	1.5 (3)
N4—N3—C8—S1	1.0 (3)	C21—C22—C23—C18	−1.2 (3)
C8—N2—C9—C10	91.8 (2)	C19—C18—C23—C22	0.0 (3)
N1—N2—C9—C10	−95.16 (19)	C17—C18—C23—C22	179.7 (2)

### Hydrogen-bond geometry (Å, °)

**Table:**

*D*—H···*A*	*D*—H	H···*A*	*D*···*A*	*D*—H···*A*
N4—H4A···O1^i^	0.91 (2)	2.39 (2)	2.873 (3)	114.(2)
N4—H4A···S1	0.91 (2)	2.69 (2)	3.194 (2)	116.(2)
N4—H4B···S1^i^	0.91 (2)	2.66 (2)	3.490 (2)	151 (2)

Symmetry codes: (i) −*x*+1, −*y*, −*z*.
